# Bromine-Lithia Water

**Published:** 1890-09

**Authors:** T. D. Crother


### BROMINE-LITHIA WATER.

A fine Lithia spring has been known for some time at a little hamlet called Lithia Springs, in Douglas county, Georgia. Recently an analysis has revealed the fact that it is the only spring known to science which contains Bromide of Potassium and Magnesia; this is combined with Lithium, Strontium and Iodide of Magnesium. The effect of this water is both a tonic and sedative, and in the army of nervous cases it gives promise of being a remedy of wonderful power. Theoretically a natural combination of the Bromides with Lithia and the Iodides would be a remedy of great value in a large number of cases. Practically, it has more than fulfilled these expectations, and although this water has been very recently introduced, there are many reasons for supposing that it will become the most widely used of any medicinal water known. Our personal experience in three cases of Alcoholic Rheumatism and Neuralgias is very satisfactory, so far, and we hope to announce in the future that at last a remedial water has been found which can be given to all Nervous Exhausted cases with great certainty as to the results. As the Hot Springs of Arkansas is the great resort of rheumatic and syphilitic cases, this Bromide Spring of Georgia may become the great resort of Neurotics of all kinds. It is perfectly clear that under any circumstances this water will become a popular remedy, and these Springs a famous resort in the near future.—T. D. CROTHER, M. D., in *Quarterly Journal of Inebriety*, for April, 1890.

